# Long-Term Effect of Maternal Antioxidant Supplementation on the Lipid Profile of the Progeny According to the Sow’s Parity Number

**DOI:** 10.3390/antiox13030379

**Published:** 2024-03-20

**Authors:** Gerardo Gómez, Hernan D. Laviano, Juan García-Casco, Maria Muñoz, Fernando Gómez, Fernando Sánchez-Esquiliche, Antonio González-Bulnes, Clemente López-Bote, Cristina Óvilo, Ana I. Rey

**Affiliations:** 1 Instituto Regional de Investigación y Desarrollo Agroalimentario y Forestal de Castilla-La Mancha (IRIAF), 13700 Toledo, Spain; g.gomez.mat@gmail.com; 2 Departamento Producción Animal, Facultad de Veterinaria, Universidad Complutense de Madrid, Avda. Puerta de Hierro s/n., 28040 Madrid, Spain; hernanlavianomvz03@gmail.com (H.D.L.); clemente@ucm.es (C.L.-B.); 3 Departamento de Mejora Genética Animal, Instituto Nacional de Investigación y Tecnología Agraria y Alimentaria (INIA), Consejo Superior de Investigaciones Científicas (CSIC), Ctra Coruña km 7.5, 28040 Madrid, Spain; garcia.juan@inia.csic.es (J.G.-C.); mariamm@inia.csic.es (M.M.); ovilo@inia.csic.es (C.Ó.); 4 Sánchez Romero Carvajal, Carretera de San Juan del Puerto, s/n, 21290 Jabugo, Spain; fernando.gomez@osborne.es (F.G.); fernando.sanchez@osborne.es (F.S.-E.); 5 Departamento de Producción y Sanidad Animal, Facultad de Veterinaria, Universidad Cardenal Herrera-CEU, CEU Universities, C/Tirant lo Blanc, 7, Alfara del Patriarca, 46115 Valencia, Spain; antonio.gonzalezbulnes@uchceu.es

**Keywords:** vitamin E, hydroxytyrosol, piglet tissues, meat quality, sow diet, antioxidants

## Abstract

Pig feeding prior to the extensive fattening phase might affect the final lipid profile and product quality. This study evaluates how maternal supplementation with vitamin E (VITE) (100 mg/kg), hydroxytyrosol (HXT) (1.5 mg/kg), or combined administration (VE + HXT) affects the piglet’s plasma and tissues’ fatty acid profiles and lipid stability according to the sow’s parity number (PN), as well as the possible changes to the lipid profile after extensive feeding. The sows’ PN affected the total fatty acid profile of plasma, muscle, and liver of piglets, with lower Δ-9 and Δ-6 desaturase indices but higher Δ-5 in those from primiparous (P) than multiparous (M) sows. Dietary VITE was more effective at decreasing C16:0 and saturated fatty acids in the muscle of piglets born from M than P sows, and modified the liver phospholipids in a different way. Sows’ supplementation with HXT increased C18:2n-6 in triglycerides and polyunsaturated fatty acids (PUFA) in muscle phospholipids. In the liver, HXT supplementation also increased free-PUFA and free-n-3 fatty acids. However, lipid oxidation of piglets’ tissues was not affected by the antioxidant supplementation, and it was higher in the livers of piglets born from M sows. The fatty acid profile in the muscle of pigs after extensive feeding was not affected by the PN, but it was by the sows’ antioxidant supplementation, with positive effects on quality by both compounds.

## 1. Introduction

During past decades, different feeding strategies that increase the content of unsaturated fatty acids, especially monounsaturated fatty acids (MUFA), to the detriment of saturated (SAT) ones have been investigated [1]. This is due to the positive relationship between the consumption of oleic acid (monounsaturated, most abundant in pork and other species) [1] and the lower risk of suffering from metabolic diseases in humans [2]. Furthermore, a higher proportion of MUFA has been associated with meat that is more stable to oxidation [3], with less production of undesirable substances and therefore higher quality [3]. MUFA can be obtained from the diet or by endogenous synthesis from SAT by the action of the delta-9-desaturase enzyme [4], which is an important component in leptin-mediated energy homeostasis, adiposity, and plasma lipid profile [5].

One of the best models of obesity and leptin resistance is the Iberian pig, which is characterized by a high MUFA intake (acorns from the Mediterranean forest) [6,7,8,9], and high desaturation capacity [10] which, as other extensive productions, result in products of high quality and price in the worldwide market [11]. This feeding under free-range conditions also provides antioxidant compounds (α and γ-tocopherols and polyphenols) that preserve the lipids layer of cell membranes [8,9,12] and the health and oxidative status of the animals [12] as well as the meat product quality [13,14,15]. Thus, this sustainable system provides products with high lipid stability and flavors and aromas that are difficult to match by other types of pig production [8,13,14,15]. 

Previous investigations indicate that certain factors such as feeding prior to the fattening phase in the Mediterranean forest under free-range conditions are decisive when it comes to obtaining an adequate antioxidant level and lipid profile of the meat [16], and therefore the quality of the final product. Thus, research carried out in fatty pigs through supplementation with antioxidants of different characteristics during growth indicates that they are in part effective at controlling oxidative stress and preserving the lipid profile and the level of other lipophilic antioxidants at the end of production [17,18]. Moreover, the antioxidant level in piglets’ tissues can be improved by maternal supplementation during lactation, mainly due to the transport of antioxidants through milk [19,20]. Recently, it has been reported that the supplementation of sows’ diets with antioxidant substances such as vitamin E (VITE) or an olive derivative like hydroxytyrosol (HXT) can significantly modify not only the antioxidant content of sows’ milk but also its lipid profile and energy intake [21], and therefore the oxidative status and growth of the piglets at birth and during the lactation period [22]. Thus, supplementing sows with VITE has shown a protective effect on desaturase enzymes and an increased ability to desaturate milk, whereas HXT increased polyunsaturated fatty acids in colostrum and seemed to inhibit desaturation [21]. The fact that a piglet receives a feed with a different degree of unsaturation could be a relevant aspect in pig production in which priority is given to obtaining final products rich in MUFA rather than polyunsaturated fatty acids (PUFA) [3,8], since the latter promote lipid oxidation and rancidity with loss of appreciation by the consumer [23]. In addition, it has been described in pigs of improved genotype that the administration of extracts derived from olive derivatives such as oleuropein during growth promotes lipid mobilization to the target tissues or metabolism of unsaturated fatty acids more quickly than the supplementation of VITE + Se [24] without altering the lipid profile of the meat [25]. However, in other studies in which olive by-products were included in the diet formulation of growing Iberian pigs before extensive feeding, some changes in SAT and MUFA were observed at slaughter [26], although the comparative effects with VITE administration were not evaluated. To the best of our knowledge, there is no information available on the long-term effects that maternal antioxidant supplementation during pregnancy or lactation have on the fatty acid profile after fattening. Also, the possible effects are unknown of the combined administration of both antioxidants (one with lipophilic characteristics such as VITE and other of hydrophobic character such as HXT) to the mother on the fatty acid profile of piglets’ tissues at the early stage and after fattening.

On the other hand, a sow’s parity number, which involves different amounts of adipose tissue reserves and mobilization of fatty acids or desaturation phenomena to obtain energy by the mother [27,28], might affected piglets’ tissues depending on the antioxidant supplementation. However, in this sense there is a complete lack of knowledge. 

It is hypothesized that antioxidant administration to a sow’s diets during gestation and lactation might modify the lipid profile of piglets’ tissues according to the mother’s parity number and may affect the final fatty acid profile after the fattening phase.

Therefore, the aims of the present study were, firstly, to evaluate how the dietary supplementation of vitamin E (VITE) (100 mg/kg), hydroxytyrosol (HXT) (1.5 mg/kg), or the combined administration (VITE + HXT) given to multiparous or primiparous Iberian sows from day 85 of gestation affects the fatty acid profiles of the plasma, muscle, and liver, and lipid stability of tissues of the offspring during the early age after weaning; and secondly, to evaluate possible changes in the fatty acid profile at slaughter after the fattening phase under extensive conditions. 

## 2. Materials and Methods

### 2.1. Chemicals

Chemicals were supplied by Sigma–Aldrich (Alcobendas, Madrid, Spain), Panreac (Castellar del Vallès, Barcelona, Spain), or Scharlau (Sentmenat, Barcelona, España) and were of analytical grade. 

### 2.2. Ethics Statement

The experiment was carried out at the animal facilities of Dehesón del Encinar (Oropesa, Toledo, Spain), which are in accordance with the requirements for Scientific Procedures Establishments. Experimental procedures related to animal handling and welfare were in compliance with the Spanish Policy for Animal Protection (RD53/2013) [29], and the European Union Directive 2010/63/UE [30] for the care and use of animals in research. The INIA Committee of Ethics in Animal Research approved the experimental procedures (report ORCEEA 2019-10).

### 2.3. Animals, Experimental Procedures and Diets

Fifty Iberian sows (half primiparous and half multiparous with between 4–5 parity) (107.2 ± 29.8 kg) that were pregnant by natural service were selected at day 85 of pregnancy for the present study (Dehesón del Encinar, Oropesa, Toledo). During the pre-experimental period sows were given a standard grain-based diet (g/kg: 888 dry matter, 124.6 protein, 29.9 fat, 49.3 fiber, 62.1 ash; and 3050 kcal/kg Metabolizable Energy) (Sanchez Romero Carvajal, Spain). At day 85 of pregnancy (126.2 ± 29.3 kg), sows were divided into four homogeneous experimental groups (12–13 per dietary treatment with equal distribution of primiparous and multiparous sows) and started receiving four different experimental diets until weaning (28 days after delivery) (Table A1). The experimental diets were supplemented with different levels of antioxidants (vitamin E or hydroxytyrosol) as follows: (1) control group received 30 mg of α-tocopheryl acetate/kg feed; (2) VITE group: 100 mg of α-tocopheryl acetate/kg; (3) HXT group: 30 mg α-tocopheryl acetate/kg and 1.5 mg hydroxytyrosol/kg, and (4) VITE + HXT: 100 mg/kg of α-tocopheryl acetate/kg + 1.5 mg hydroxytyrosol/kg feed. Feed administration was adjusted to fulfill daily maintenance requirements according to the National Research Council [26] from day 85 of gestation until day 28 of lactation (experimental period). To reach the α-tocopherol levels recommended by the NRC [31] for sows, a dose of 30 mg α-tocopherol/kg feed was used for basal diet supplementation. The α-tocopheryl acetate used in the diets was purchased from DSM Nutritional Products (Alcalá de Henares, Madrid, Spain) and the hydroxytyrosol extract (Olea europaea L. dry extract, N20130102) was obtained from Natac (Alcorcón, Madrid, Spain). The supplier certified that the HXT extract contained a minimum of 1.5% (Natac, Alcorcón, Madrid, Spain).

The piglets (average litter size of 7–8) were injected with intramuscular iron behind the ear (Previron 200, Hipra, Talavera de la Reina, Toledo, Spain) a few hours after birth (first 24 h) to avoid anemia problems. In addition, their tails were cut off and they were marked with ear tags for easy identification. All facilities and management were in compliance with RD 53/2013 [29]. Piglets remained with the sows until weaning. 

### 2.4. Sample Collection

Five days after weaning (day 33), 48 male piglets (1 per litter; n = 12 per dietary treatment) from a total of 344 were selected at random (6.1 ± 1.3 kg) and euthanized in compliance with RD 53/2013. Then, longissimus dorsi and liver samples were collected, placed inside a plastic bag, transported in dry ice, and kept at −80 °C until analysis. 

The piglets that were not slaughtered after weaning (n = 296) followed the growth and fattening phases. During growth, all pigs received the same feed, which was based on formulated diets according to FEDNA [32] recommendations for Iberian pigs (Table A2). During the growth period, pigs were maintained in open boxes at an average temperature of 20 ± 5 °C and humidity of 58 ± 13%. 

The fattening consisted of free-range feeding with acorns and grass in a Mediterranean forest (Deheson del Encinar, Toledo, Spain) according to the traditional practice for Iberian pigs in Spain [8,9], until approximately 15 months of age (February 2021), after which they were sent to a commercial slaughterhouse with an average weight of 164.22 ± 20.47 kg (Sanchez Romero Carvajal, Jabugo, Huelva, Spain). During this fattening phase, the pigs were kept at an environment temperature of 10 ± 4 °C and humidity of 80 ± 5%. 

At the time of slaughter, a small piece of longissimus dorsi muscle was taken for analysis. Samples were kept in plastic bags, transported on ice, and kept at −80 °C until analysis.

### 2.5. Laboratory Analysis

#### 2.5.1. Fat Content and Fatty Acid Profile of Plasma, Muscle, and Liver Samples

Total lipids of plasma, muscle, or liver were extracted as described elsewhere [24,25], and then analyzed for fatty acid profile determination. Freeze-dried samples (Lyoquest, Telstar, Tarrasa, Spain) were weighted in an Eppendorf, and a solvent mixture of dichloromethane–methanol 8:2 was added. After homogenization in a mixer mill (MM400, Retsch technology, Haan, Germany) and centrifugation for 8 min at 10,000 rpm (Hermle Z383-K; Wehingen, Germany), the upper layer containing lipids was collected. The lipid content was quantified gravimetrically after evaporation of the solvent in a nitrogen stream. 

Neutral lipids (NL)—mainly triglycerides, free fatty acids (FFA) and polar lipids (PL)— in muscle and liver samples were separated from the total lipid extracts using aminopropyl minicolumns (Varian, Harbor City, CA, USA), which had previously been activated with hexane (7.5 mL) [24,25]. After fat addition dissolved in hexane:chloroform:methanol, (95:3:2), NL were extracted with chloroform (5 mL), FFA with diethylether:acetic acid (98:2) (5 mL), and PL with chloroform:methanol (1:6) followed by chloroform:sodium acetate (0.05 M in methanol) (1:6). 

Fatty acid methyl esters (FAMEs) were obtained by the addition of methanol:toluene: H_2_SO_4_ (88:10:2 by volume) after heating the lipids (80 °C for 1 h) [33]. Then, FAMEs were extracted with hexane and separated after sample injection in a gas chromatograph (HP 6890 Series GC System; Hewlett Packard, Avondale, PA, USA). The gas chromatograph was provided with an automatic injector, a capillary column (HP-Innowax polyethylene glycol, 30 m × 0.316 mm × 0.25 µm), and a flame ionization detector (hold at 250 °C). After injection (1 µL), the oven temperature was increased from 170 °C to 210 °C at a rate of 3.5 °C/min, then to 250 °C at a rate of 7 °C/min [24]. Identification and quantification of the FAMEs were made by comparing the retention times with those of authentic standards (Sigma–Aldrich, Alcobendas, Spain). The results were expressed as grams per 100 g of quantified fatty acids. 

The ∆-9, ∆-6, and ∆-5- desaturase indices were calculated as described elsewhere [21] using the formulas:

Δ9 − desaturase index = (C14:1n-5 + C16:1n-7 + C18:1n-9 + C18:1n-11)/(C14:0 + C14:1n-5+ C16:0 + C16:1n-7 + C18:0 + C18:1n-9 + C18:1n-11)

Δ5-desaturase = (C20:4n-6)/(C20:3n-6+C20:4n-6)

Δ6-desaturase = (C18:3n-6 + C18:4n-3)/(C18:2n-6 + C18:3n-3 + C18:3n-6 + C18:4n-3)

#### 2.5.2. Tocopherol Quantification in Longissimus Dorsi Muscle and Liver Samples

The α-tocopherol (vitamin E) concentration in muscle and liver samples from weaned piglets was quantified by direct extraction following the procedure reported by Rey et al. [9]. Different volumes of ethanol were added to duplicate muscle or liver samples followed by homogenization (5 min at 30 Hz in a Mixer Mill MM400, Retsch technology, Haan, Germany), to a final volume of 10 mL. Tocopherol was extracted by centrifugation at 10,000 rpm for 5 min at 4 °C (Hermle Z383-K; Wehingen, Germany) and the supernatant was collected and evaporated by an N_2_ stream. The remaining residue was dissolved in ethanol and injected into an HPLC (HP 1200, equipped with a diode array detector and a reverse RP-18 column) (Agilent Technologies, Waldbronn, Germany) [9]. A calibration curve was built with the pure compound (Sigma–Aldrich, Alcobendas, Madrid) that allowed its identification by the retention peak and its quantification. The results were expressed as µg of α-tocopherol per g of muscle or liver.

#### 2.5.3. Iron-Induced Lipid Oxidation in Muscle and Liver Samples

The liability of muscle and liver homogenates to iron ascorbate-induced lipid oxidation was determined by a modification of the method of Kornbrust and Mavis [34]. Homogenates (approximately 1 mg protein/mL) were incubated at 37 °C in 40 nM Tris-maleate buffer (pH 7.4) with 1 mM FeSO_4_ in a total volume of 10 mL in duplicated samples. At fixed intervals (0, 60, 90, and 120 min), 0.4 mL were removed for measurement of 2-thiobarbituric acid-reactive substances (TBARs). Absorbance was measured spectrophotometrically at 532 nm (ScanGo, ThermoFisher Scientific, Alcobendas, Spain). TBARS were expressed as nmol malondialdehyde (MDA)/kg meat or liver. A standard curve was built with 1,1,3,3-Tetraethoxipropane.

### 2.6. Statistical Analysis

For analysis of data, the general linear model (GLM) procedure contained in SAS (version 9.4; SAS Inst. Inc., Cary, NC, USA) was used, and included the fixed effects of VITE vs. HXT and sows’ parity numbers (multiparous versus primiparous) with their interaction in a full factorial model. The individual piglet was considered the experimental unit. Comparison between means was conducted using the Duncan test. Data are presented as the mean of each group and root mean square error (RMSE) together with the significance levels (*p* value). Differences were considered statistically significant when *p* < 0.05, whereas *p* > 0.05 and <0.1 was considered as a trend. 

## 3. Results

### 3.1. Total Fatty Acid Profile of Plasma, Longissimus Dorsi Muscle, and Liver of Piglets

**Figure 1:**
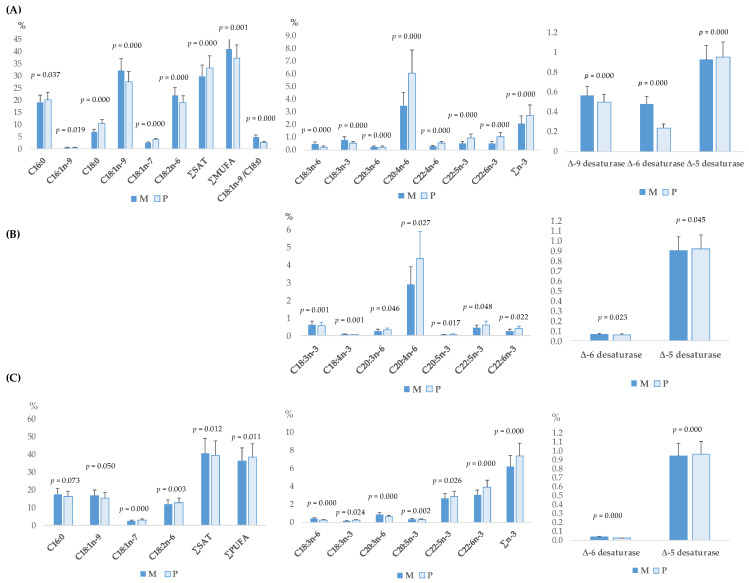
Significant effect of the maternal parity number (multiparous: M; or primiparous: P), on the total fatty acid profile of plasma (**A**), longissimus dorsi muscle (**B**), or liver (**C**) of piglets 5 days post-weaning. ∑SAT = sum of total saturated fatty acids; ∑MUFA = sum of total monounsaturated fatty acids; ∑PUFA = sum of total polyunsaturated fatty acids; Δ-9−desaturase index = (C14:1 + C16:1 + C18:1)/C14:0 + C14:1 + C16:0 + C16:1 + C18:0 + C18:1); Δ6-desaturase = (C18:3n-6 + C18:4n-3)/(C18:2n-6 + C18:3n-3 + C18:3n-6 + C18:4n-3); Δ5-desaturase = (C20:4n-6)/(C20:3n-6 + C20:4n-6).

The significant fatty acids of the plasma, muscle, or liver of weaned piglets according to the sow’s parity number are presented in Figure 1. Piglets from multiparous sows had greater proportions of C16:0 (*p* < 0.001), C18:0 (*p* < 0.001), ∑SAT (*p* < 0.001), C20:4n-6 (*p* < 0.001), C22:4n-6 (*p* < 0.001), C22:5n-3 (*p* < 0.001), C22:6n-3 (*p* < 0.001), total ∑n-3 (*p* < 0.001), and Δ-5-desaturase index (*p* < 0.001) in plasma that those from primiparous sows. However, piglets from multiparous sows had lower C16:1n-9 (*p* < 0.001), C18:1n-9 (*p* < 0.001), ∑MUFA (*p* < 0.001), C18:1/C18:0 (*p* < 0.001), C18:3n-6 (*p* < 0.001), C18:3n-3 (*p* < 0.001), C20:3n-6 (*p* < 0.001), and Δ-9 and Δ-6-desaturase indices (*p* < 0.001) than from primiparous sows in plasma. Similar results were observed in many of the polyunsaturated fatty acids (∑PUFA) in the longissimus dorsi muscle of the piglets. In the liver, a similar trend to plasma and muscle was observed for the C18:1n-9, C18:3n-6, C20:3n-6, C22:5n-3, C22:6n-3, ∑n-3, Δ-5, and Δ-6 desaturases. On the contrary, in the liver, C16:0 (*p* < 0.01) and ∑SAT (*p* < 0.05) decreased whereas C18:2n-6 (*p* < 0.01) and ∑PUFA (*p* < 0.05) increased.

No significant changes were observed according to dietary effect in the total lipid fraction of plasma or different tissues. Neither the interaction between the parity number or dietary treatment was found for the total lipid fraction. 

### 3.2. Fatty Acid Fractions of Longissimus Dorsi Muscle and Liver of Piglets

**Figure 2:**
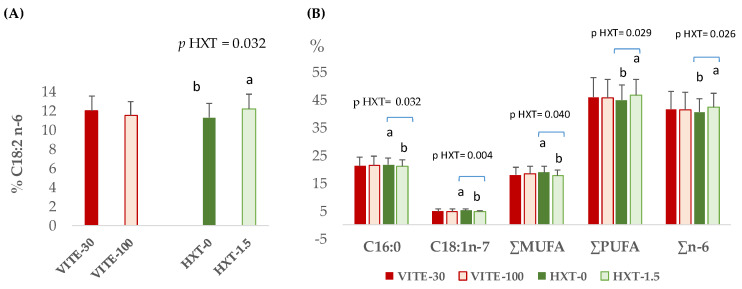
Significant effects of the maternal supplementation with VITE (30 vs. 100 mg α-tocopherol acetate/kg) or HXT (0 vs. 1.5 mg/kg) on the fatty acid profile (**A**) triglycerides or (**B**) phospholipids of longissimus dorsi muscle from piglets 5 days post-weaning. ∑MUFA = sum of total monounsaturated fatty acids; ∑PUFA = sum of total polyunsaturated fatty acids. ^a,b^ Numbers with different superscript were statistically significant.

The different fatty acid fractions (triglycerides and phospholipids) of the longissimus dorsi muscle from piglets post-weaning according to the dietary supplementation of the sows (VITE or HXT) are presented in Figure 2. 

Sows’ dietary supplementation with VITE did not modify the triglycerides or phospholipid fractions of piglets’ muscle. However, the supplementation of sows with HXT produced an increase in the C18:2n-6 (*p* = 0.032) proportion of muscle triglycerides (Figure 2A). Also, sows’ HXT supplementation modified the phospholipid fraction of piglets’ muscle. Thus, the proportion of ∑PUFA (*p* = 0.029) and ∑n-6 (*p* = 0.026) increased in piglets from sows supplemented with HXT compared to those without supplementation, whereas the proportion of C16:0 (*p* = 0.032), C18:1n-7 (*p* = 0.004), and ∑MUFA (*p* = 0.040) decreased (Figure 2B).

**Figure 3:**
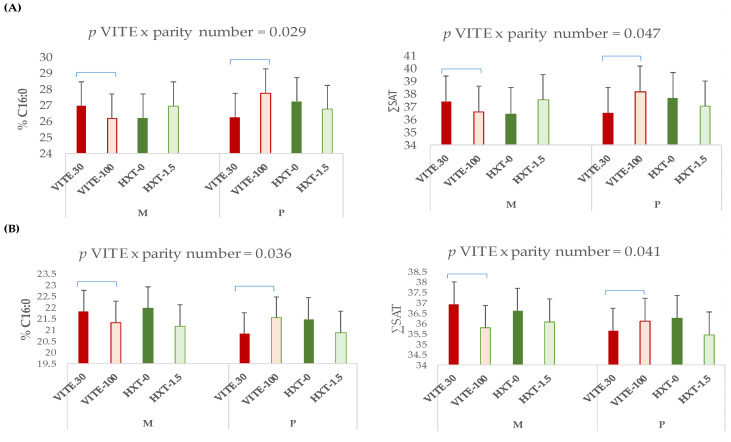
Interaction effect between the maternal parity number (multiparous: M; or primiparous: P) and the antioxidant supplementation on the fatty acid profile of (**A**) triglycerides or (**B**) phospholipids of longissimus dorsi muscle from piglets 5 days post-weaning. ∑SAT = sum of total saturated fatty acids.

No interactions between VITE and HXT supplementation of the sow were observed on the muscle lipid composition of the piglet. However, a qualitative interaction effect between the maternal parity number and the VITE supplementation was observed on the fatty acid profile of triglycerides or phospholipids in the longissimus dorsi muscle from piglets 5 days post-weaning (Figure 3).

Thus, the supplementation of the sow with VITE decreased the C16:0 (*p* = 0.029) and ∑SAT (*p* = 0.047) in multiparous sows compared to primiparous sows, and these last ones presented a clear increase of these fatty acids in triglycerides and phospholipids of muscle. 

No interactions were observed between the HXT supplementation of the mother and the parity number on the different muscle fatty acid fractions of piglets. 

**Figure 4:**
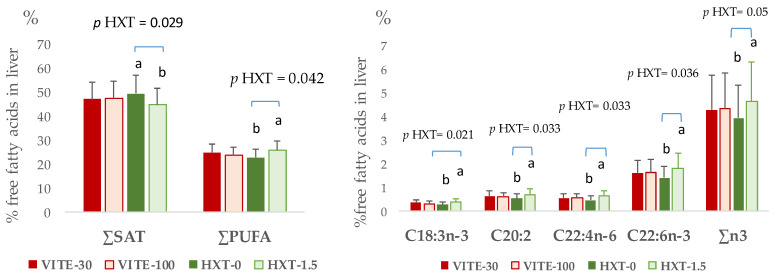
Significant effects of maternal supplementation with VITE (30 vs. 100 mg α-tocopherol acetate/kg) or HXT (0 vs. 1.5 mg/kg) on the free fatty acid profile of liver from piglets 5 days post-weaning. ∑SAT = sum of total saturated fatty acids; ∑PUFA = sum of total polyunsaturated fatty acids. ^a,b^ Numbers with different superscript were statistically significant.

The effect of maternal supplementation with antioxidants on hepatic tissue is presented in Figure 4. No changes according to the dietary antioxidant supplementation were observed on the triglyceride or phospholipid fractions of liver samples. However, antioxidant supplementation of the mother modified other fractions of fatty acids such as free-fatty acids (Figure 4). Thus, the sow’s HXT supplementation increased the proportion of free ∑PUFA (*p* = 0.042), C18:3n-3 (*p* = 0.021), C20:2 (*p* = 0.033), C22:4n-6 (*p* = 0.033), C22:6n-3 (*p* = 0.036), and ∑n-3 (*p* = 0.050), whereas the proportion of ∑SAT decreased (*p* = 0.029). 

**Figure 5:**
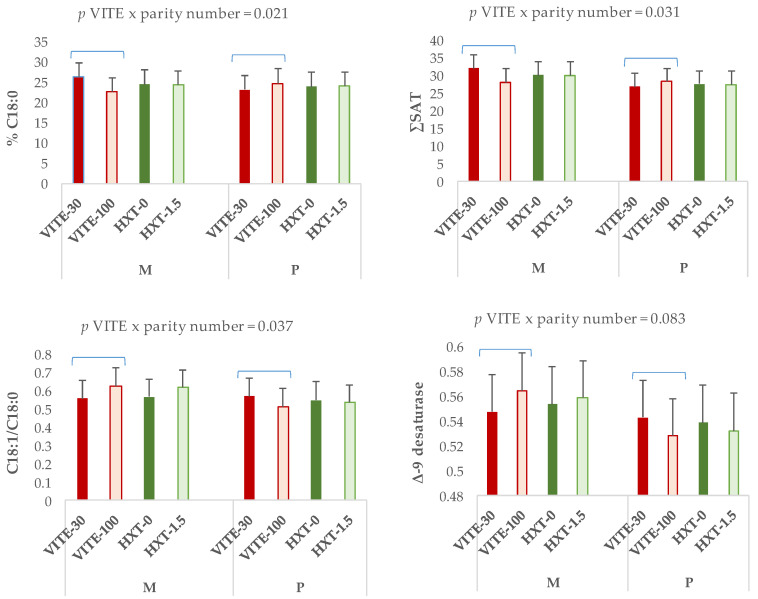
Interaction effect between the maternal parity number (multiparous: M; or primiparous: P) and antioxidant supplementation on the fatty acid profile of phospholipids of the liver from piglets 5 days post-weaning. ∑SAT = sum of total saturated fatty acids; Δ-9−desaturase index = (C14:1 + C16:1 + C18:1)/C14:0 + C14:1 + C16:0 + C16:1 + C18:0 + C18:1).

VITE supplementation did not modify any of the fractions of piglets’ liver. However, an interaction effect was observed between the maternal parity number and the VITE supplementation on the fatty acid profile of liver phospholipids of piglets (Figure 5). Thus, piglets from multiparous sows supplemented with 100 mg/kg of VITE presented lower proportions of C18:0 (*p* = 0.021) and ∑SAT (*p* = 0.031), but higher C18:1n-9/C18:0 (*p* = 0.037) compared to primiparous sows. Also, VITE supplementation tended to increase Δ-9-desaturase activity (*p* = 0.083) in piglets’ liver from multiparous sows but to a lesser extent in primiparous sows. 

No interaction effects were observed between the parity number of the sow and the HXT supplementation on the liver phospholipids of piglets. 

### 3.3. Lipid Stability in Tissues of Weaned Piglets

**Figure 6:**
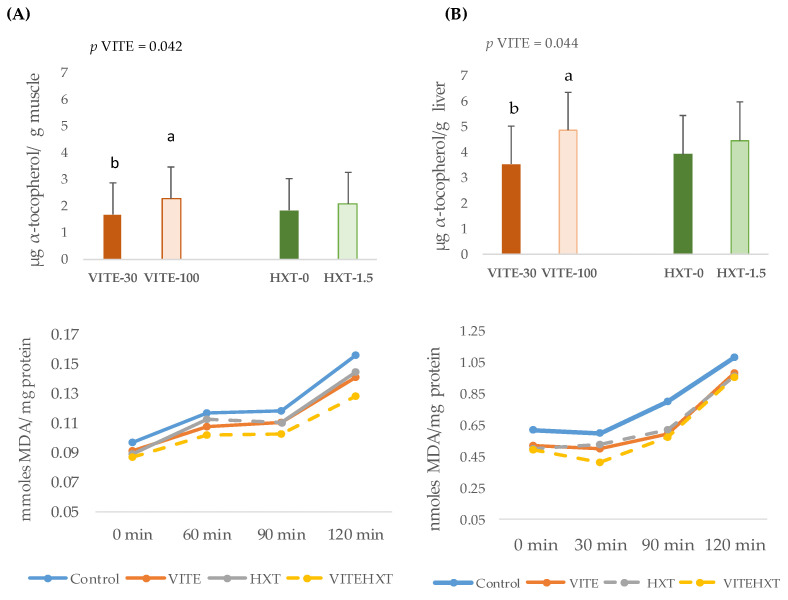
Effects of the sows’ supplementation with VITE (30 vs. 100 mg α-tocopherol acetate/kg) or HXT (0 vs. 1.5 mg/kg) on the α-tocopherol content and MDA concentration of muscle (**A**) or liver (**B**) from piglets 5 days post-weaning. Control = 30 mg of α-tocopheryl acetate/kg feed + 0 mg/kg hydroxytyrosol; VE = 100 mg of α-tocopheryl acetate/kg feed + 0 mg/kg hydroxytyrosol; HXT = 30 mg of α-tocopheryl acetate/kg feed + 1.5 mg/kg hydroxytyrosol; VE + HXT = 100 mg of α-tocopheryl acetate/kg feed + 1.5 mg/kg hydroxytyrosol; *p* = differences were statistically different when *p* < 0.05; ^a,b^ Numbers with different superscripts were statistically significant.

The α-tocopherol concentration and lipid stability of the longissimus dorsi muscle or liver of weaned piglets according to the dietary supplementation of the sow are presented in Figure 6. 

Some changes were observed in the α-tocopherol concentration of both tissues. Thus, the piglets from sows supplemented with VITE presented a higher concentration of α-tocopherol in the longissimus dorsi muscle (*p* = 0.042) and liver (*p* = 0.044) (Figure 6A,B). HXT supplementation of the sow did not modify the α-tocopherol content in muscle or liver (*p* > 0.05). Neither an interaction effect between sows’ VITE nor HXT supplementation was observed on the tocopherol content of piglets’ tissues. 

On the other hand, the MDA concentrations of piglets’ muscle and liver were not statistically modified by the sows’ antioxidant supplementation, and no interaction effect of either antioxidant was observed. 

**Figure 7:**
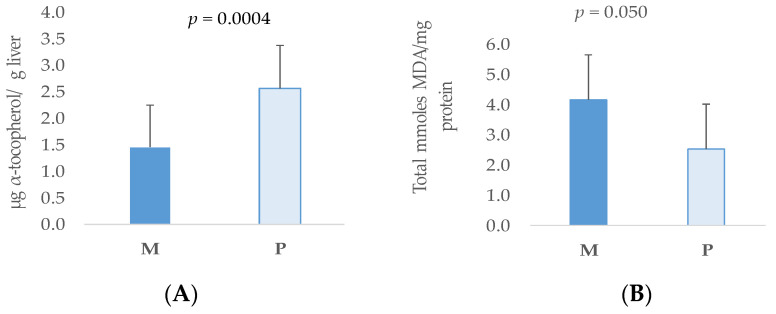
Significant effect of the maternal parity number (multiparous: M; or primiparous: P) on the alpha-tocopherol content (**A**) and malondyaldehyde (MDA) concentration (**B**), in liver samples from piglets 5 days post-weaning.

Concerning a sow’s parity number, no effect or interactions with the antioxidant supplementation on the concentration of α-tocopherol or total TBARs were observed in muscle. However, in piglets’ liver, the concentration of α-tocopherol was greater in those born from primiparous sows than from multiparous sows (*p* = 0.0004) (Figure 7A). 

On the contrary, the concentration of MDA was higher in the liver of piglets born from multiparous sows than from primiparous sows (*p* = 0.050) (Figure 7B). 

No interactions were observed between antioxidant supplementation and the parity number on the α-tocopherol or MDA concentrations in the liver of piglets.

### 3.4. Final Weights and Carcass Characteristics of Pigs after Fattening 

The final weights and carcass characteristics of pigs after the fattening phase according to the antioxidant supplementation are presented in Table 1. No changes were observed in any of the variables studied, and neither the weight of the carcass nor the weight of the main pieces (ham and loin) were affected by the sows’ supplementation with antioxidants or by the parity number.

### 3.5. Fatty Acid Profile of Intramuscular Fat of Muscle after Fattening under Free-Range Conditions

The fatty acid profile of intramuscular fat from pigs after the fattening phase is presented in Table 2. 

The pigs from sows supplemented with VITE presented higher proportions of C16:1n-7 (*p* = 0.031) and ∑MUFA (*p* = 0.047), and tended to have a higher Δ-6-desaturase index (*p* = 0.070). However, these groups had lower proportions of C16:1n-9 (*p* = 0.025), C18:2n-6 (*p* = 0.038), ∑n-6 (*p* = 0.031), ∑n-6/∑n-3 ratio (*p* = 0.008), and ∑PUFA (*p* = 0.033). 

On the other hand, pigs from sows supplemented with HXT had higher proportions of C18:3n-3 (*p* = 0.037) and ∑n-3 (*p* = 0.018), and lower ∑n-6/∑n-3 ratios (*p* = 0.038). No interaction effect between the administrations of either antioxidant was observed on the fatty acid profile of the intramuscular fat. 

Since neither parity number effect nor interactions of parity number and antioxidant supplementation were observed, these data are not presented in Table 1 and Table 2.

## 4. Discussion

The piglets’ tissues and final fatty acid profile after the extensive fattening phase might be affected by changes in the degree of unsaturation and oxidation of milk due to sows’ antioxidant supplementation during lactation [21]. In addition, the fact that sows had different energy demands depending on the parity number could also affect the starting fatty acid profile and to a certain extent the fatty acid transfer and final composition of the piglets’ tissue. However, there is no information on how different antioxidants or their combination modify tissue lipid accumulation in piglets and whether these can act to different extents depending on the age of the sow.

According to the results of the present study, a sow’s parity number significantly affects the tissue lipid profile of piglets with less ∑MUFA and more ∑SAT, as well as less ∆-9 and ∆-6 desaturase capacity in those that came from primiparous mothers; however, ∆-5 desaturase showed the opposite effect with a greater proportion in those from primiparous mothers. Age-related changes in desaturation have been observed previously [35]. Thus, in rats, liver ∆-6 desaturation increased with age, whereas ∆-5 decreased markedly from the first period of life [35]. In the specific case of the pig, changes have also been observed with age and development [36], and a decrease in ∆-5 during growth has been reported [37]. ∆-9 desaturase and ∑MUFA have also showed a direct correlation with the fat content of the muscle, whereas ∆-5 and ∑PUFA were negatively correlated [37], which is in accordance with the differences observed in the present study in piglets from older and heavier (multiparous) versus younger and lighter sows. This is of interest because some studies indicate that stearoyl-CoA desaturase deficiency activates metabolic pathways that promote β-oxidation and decrease lipogenesis [38]. To our knowledge there is no further information in the literature concerning the effect of the parity number on piglets’ tissue composition, and this study provides very novel information. Therefore, the fact that the sows were in different development states and nutrient and energy needs as well as lipid reserves during lactation could affect their fatty acid metabolism and fatty acid transfer through milk to the piglets, and modify the piglets’ lipid metabolism. 

On the other hand, although the piglets’ total lipid fractions of plasma, muscle, and liver were not modified by the antioxidant supplementation of the sow, a detailed study of the different lipid fractions showed some interesting changes. In intramuscular fat, the phospholipid fraction was the most affected, followed by triglycerides. Thus, HXT supplementation to sows’ diets increased the proportion of C18:2n-6 in triglycerides of piglets’ muscle and the ∑PUFA (mainly n-6) also increased in phospholipids, which resulted in lower ∑MUFA and C16:0. This effect was in accordance with higher values of PUFA in the milk of mothers who received 1.5 mg/kg HXT [21] and as a result of its transfer to the piglet since only 5 days passed after weaning until sampling. However, these higher levels of C18:2 were not maintained over time, and 15 months after the fattening period in free-range conditions, the intramuscular fat did not show changes in the proportion of this fatty acid. This result is interesting for the quality of meat since the linoleic acid proportion is associated with obtaining a negative flavor and greater rancidity due to easier oxidation and hexanal production [39,40]. Concerning the effects observed in other studies on this fatty acid by HXT supplementation, Dias et al. [41] found a decrease in C18:2 proportion in breast meat from broilers according to the increase in the dose of HXT. However, other authors did not find differences in the proportion of this fatty acid in pork of lean genotype supplemented with oleuropein and subjected to fasting and stress prior to slaughter [25], although plasma levels of C18:2 and PUFA were lower compared to the control group [24]. This effect was attributed to faster uptake of circulating glucose and lipolysis initiation in this group of pigs [24]. In vitro studies show an inhibitory effect of oleuropein and hydroxytyrosol on lipid synthesis, and an increase in lipid oxidation [42], and recent investigations confirm that HXT induces systemic dyslipidemia in humanized mice [43]. It is of interest to remark that all these studies were developed with direct supplementation of HXT in the growing–fattening phase, without information being available on the long-term effects of maternal supplementation on the tissue composition of the piglet. The drop in intramuscular C18:2 levels over time in the group from HXT-supplemented sows could be in part attributed to the greater consumption of foods rich in MUFA (acorns) under extensive conditions, but also to a preferential and more rapidly use of C18:2 for metabolic purposes (locomotion and thermoregulation costs under the cold winter) over other SAT or MUFA [44]. It has been observed that the higher the levels (as observed in piglets from the HXT group), the greater its use [45] but also the oxidation for energy could be kept faster in piglets from HXT-supplemented sows as it happens in the mother. An increased DNA methylation and epigenetic regulation has been observed in fetuses from sows supplemented with HXT in previous studies, although the fetuses were not permanently exposed to hydroxytyrosol [46].

On the contrary, n-3 fatty acids were not significantly affected in the muscle of piglets by the supplementation of the mother with HXT, although in fact the day-20 milk presented higher proportions of C18:3n-3 in this group. This result seems opposite to what was observed by other authors [24] in which, as mentioned, HXT would enhance the mobilization of PUFA by the sow, and it could indicate a very fast utilization of this type of fatty acid by the piglets. However, these fatty acids did increase after extensive feeding in HXT-supplemented pigs compared to those from sows that did not receive HXT supplementation, and consequently the n-6/n-3 ratio was reduced in meat. It is important to highlight that the Iberian pig fattened in extensive conditions occasionally consumes a good amount of grass that increases n-3 deposits in fat [6,7,8,9]. The fact that piglets from sows that received HXT had higher levels of the same fatty acids (mainly C18:3n-3) after fattening might be attributed to changes in ∆-5 desaturase activity due to the action of sows’ supplementation with HXT. Mothers supplemented with HXT did present a lower ∆6 + ∆5 capacity in day-20 milk [21] and this inhibitory effect on desaturases could be maintained by the piglets for a time after weaning, although changes in desaturase indices of piglets’ muscle did not reach statistical significance and only a tendency was observed. In an interesting review carried out by Mennitti et al. [47], it was reported that epigenetic alterations that occur during development are controlled through the transcriptional regulation of fatty acid desaturases in the offspring.

The study of the lipid profile of the piglets’ livers supported the results obtained in the muscle in such a way that greater mobilization and metabolic utilization would produce a greater proportion of free fatty acids, as observed in the liver of the present study. Thus, HXT supplementation of the sow produced an increase in the fraction of free-PUFA fatty acids in the liver tissue of the piglets. Specifically, the increase was mainly in free n-3 fatty acids, followed by long-n-6 fatty acids and would confirm their faster utilization for different purposes. Similar results were observed in previous investigations in fattened pigs [24,25] but the maternal effect was not studied. Therefore, this result would again indicate that the metabolic changes induced in the sow by the action of HXT would be maintained in the piglet, despite the limited transfer and accumulation of HXT [46].

On the contrary, VITE supplementation to the sow did not seem to alter the lipid profile of the different fractions in piglets’ tissues, despite the changes found in colostrum and milk in multiparous sows [21]. However, it was evident that VITE supplementation changed the fatty acid profile of piglets’ muscle depending on the sows’ parity number (interaction effect) and a clear decrease in C16:0 and SAT was observed in the piglets from multiparous sows supplemented with 100 mg/kg VITE compared to those that received the basal dose of 30 mg/kg, whereas the opposite effect was observed in primiparous sows. This is the first study in which the combined effect of a sow’s parity number and its supplementation with VITE on the tissue composition of the piglet is evaluated. Changes in SAT content of piglets’ muscle could be attributed to the positive effects of VITE on Δ-9 desaturase activity [48,49], which, as indicated above, were more evident at older ages and development stages [36,37]. Hence, it has been observed that the activity of desaturases may be regulated during aging to maintain the level of PUFA in membranes [35].

These results were confirmed in the liver tissue of the piglets with even more marked effects on ∆-9 in those from multiparous mothers that received higher doses of VITE. A positive relation between VITE concentrations and the activity of different desaturases and elongases has previously been described [48,49,50]. Vitamin E was efficiently transferred to the tissues, both muscle and liver, where it could have exerted its antioxidant action [19,20,22] and could protect desaturase enzymes, which are very susceptible to oxidation [51], enhancing their effect. Moreover, some vitamin E metabolites have been reported to act as essential enzyme cofactors for the mitochondrial desaturation–elongation pathway [52]. In fact, in the present study, multiparous sows had lower VITE values in the liver than primiparous sows, which could be due to their greater expenditure exerting its actions. Other authors have reported that primiparous cows present higher antioxidant status than multiparous cows [53].

An aspect of special relevance in the present study is that although the effects of VITE on the capacity to desaturate were initially greater in piglets from multiparous sows, after fattening, no such change due to the number of farrowings was observed, but the effect of VITE was maintained over time. Thus, pigs from this group had higher MUFA and lower C18:2n-6, and tended to have higher ∆-6 desaturase in muscle that resulted in lower n-6/n-3. Modifications have been described in the desaturase activity in obesity [54], so changes in weight or body composition could affect these enzymes. However, the sows’ performances and piglets’ weights after weaning were not statistically affected by sows’ antioxidant supplementation [22], and slaughter weights were not affected either by any of the effects evaluated in the present research. Thus, differences in fatty acids after the fattening phase could not be attributed to the type of birth. Since all piglets received the same feeding with basal levels of VITE during growth, these results would again indicate a long-term effect of maternal antioxidant supplementation. Other authors have described epigenetic changes due to the use of VITE in the mother’s diet [55], although there is hardly any information available.

This is also an interesting result from the point of view of meat quality since in pork it is difficult to achieve an n-6/n-3 balance that meets the recommendations for consumer health [40], so any improvement at this level represents an advance. Furthermore, one of the objectives when establishing measures to improve quality in Iberian pig production is to achieve high proportions of MUFA and low PUFA in meat products [7,8,13], so supplementing gestating sows with VITE would be an interesting strategy.

Taking into account the modifications in the lipid profile of the piglets’ tissues, changes in oxidative stability would be expected. Thus, according to this study, piglets from HXT-supplemented sows might experience higher peroxide and hydroperoxide formation since these are formed once the free radicals are produced by the presence of oxygen [56]. The formation of primary and secondary by-products has been proposed to have implications on meat quality and human health [56]. Thus, lipid oxidation may modify flavor and color and reduce the shelf-life of meat [57]. However, the lipid stability of piglets’ tissues from mothers supplemented with antioxidants did not present significant changes, although the MDA concentration was numerically lower in piglets from mothers that received higher antioxidant supplementation. It has been widely studied that at higher doses of VITE supplementation in the feed, greater effects on the oxidation stability of tissues are observed [19,20]. However, in the present study, the difference in supplementation doses between the basal and supplemented groups (30 vs. 100) was less marked than in observations from other authors who used higher doses [17,20]. On the other hand, HXT did not modify VITE concentrations in the tissues at weaning and it does not seem to accumulate [46] in the same way as vitamin E in the tissues, which would explain the lack of marked effects at this level.

## 5. Conclusions

In conclusion, fatty acid profile in piglets’ tissues and desaturase activity changed according to the parity number of the sows. The administration of 1.5 mg/kg HXT to sows from late gestation increased the PUFA (mainly C18:2) proportion of triglycerides and phospholipids of intramuscular fat, as well as the proportion of free-PUFA (mainly n-3) in the livers of weaned piglets. However, 100 mg/kg of VITE supplementation to sows’ diets increased the capacity to desaturate in weaned piglets from multiparous mothers but not in those from primiparous mothers, and changes were maintained over time. Thus, the meat of fattened pigs from VITE-supplemented sows had higher MUFA, and lower PUFA (mainly C18:2) and n-6/n-3, which would indicate that this is an interesting strategy to improve the meat’s lipid profile. The use of HXT did not produce such marked changes after fattening, but no negative effects were observed on the lipid profile of the meat.

## Figures and Tables

**Table 1:** Effect of maternal supplementation with VITE (30 vs. 100 mg α-tocopherol acetate/kg) or HXT (0 vs. 1.5 mg/kg) on the final weight and carcass characteristics of Iberian pigs at slaughter (15 months of age) after free-range fattening.

	VITE-30	VITE-100	HXT-0	HXT-1.5	RMSE ^1^	*p* VITE ^2^	*p* HXT	*p* VITEXHXT
**Body weight, kg**	163.46	166.63	163.92	166.17	20.355	0.105	0.544	0.113
**Carcass weight, kg**	129.74	132.59	130.24	132.09	16.197	0.106	0.586	0.134
**Left ham weight, kg**	10.69	10.61	10.50	10.81	1.042	0.837	0.289	0.989
**Right ham weight, kg**	10.78	10.79	10.65	10.91	1.084	0.812	0.414	0.796
**Left loin weight, kg**	1.55	1.54	1.53	1.56	0.169	0.988	0.372	0.903
**Right loin weight, kg**	1.55	1.53	1.52	1.56	0.168	0.541	0.182	0.624

^1^ RMSE = Root mean square error (pooled SD); ^2^ *p* = differences were statistically different when *p* < 0.05.

**Table 2:** Effect of maternal supplementation with VITE (30 vs. 100 mg α-tocopherol acetate/kg) or HXT (0 vs. 1.5 mg/kg) on the total fatty acid profile of intramuscular fat of the longissimus dorsi muscle (%) from Iberian pigs at slaughter (15 months of age) after free-range fattening.

	VITE-30		VITE-100		HXT-0		HXT-1.5		RMSE ^1^	*p* VITE ^2^	*p* HXT	*p* VITEXHXT
**IMF ^3^**	12.842		12.151		12.727		12.266		6.216	0.5518	0.6912	0.9136
**C14:0**	1.465		1.476		1.447		1.493		0.257	0.8241	0.3406	0.1426
**C15:0**	0.034		0.033		0.033		0.034		0.011	0.8050	0.5973	0.9736
**C16:0**	22.628		22.777		22.862		22.544		2.058	0.6969	0.4078	0.2493
**C16:1n-9**	0.257	a	0.237	b	0.245		0.249		0.046	0.0246	0.6141	0.0846
**C16:1n-7**	3.691	b	3.967	a	3.853		3.805		0.680	0.0311	0.7045	0.7540
**C17:0**	0.163		0.157		0.155		0.164		0.038	0.3931	0.2039	0.6529
**C17:1**	0.244		0.245		0.250		0.239		0.071	0.9098	0.4183	0.9598
**C18:0**	10.920		10.589		10.718		10.790		1.285	0.1685	0.7653	0.1592
**C18:1n-9**	46.239		47.077		46.425		46.892		2.468	0.0706	0.3116	0.1830
**C18:1n-7**	4.827		4.754		4.859		4.722		0.855	0.6445	0.3914	0.7541
**C18:2n-6**	6.167	a	5.498	b	5.832		5.834		1.716	0.0384	0.9947	0.8210
**C18:3n-6**	0.045		0.041		0.044		0.042		0.016	0.1398	0.5532	0.9296
**C18:3n-3**	0.195		0.194		0.185	b	0.205	a	0.050	0.9097	0.0372	0.7318
**C18:4n-3**	0.085		0.087		0.084		0.088		0.016	0.5937	0.1830	0.2434
**C20:0**	0.156		0.151		0.152		0.154		0.031	0.3786	0.7754	0.3473
**C20:1n-9**	0.820		0.832		0.819		0.833		0.101	0.5210	0.4487	0.0663
**C20:2n-6**	0.212		0.200		0.199		0.213		0.051	0.2072	0.1452	0.6099
**C20:3n-6**	0.102		0.095		0.098		0.098		0.028	0.1659	0.9285	0.3311
**C20:4n-6**	1.750		1.591		1.740		1.601		0.507	0.0936	0.1433	0.7696
**∑SAT ^4^**	35.365		35.182		35.368		35.179		2.709	0.7177	0.7082	0.0924
**∑MUFA ^5^**	56.078	b	57.112	a	56.450		56.740		2.774	0.0474	0.5757	0.1462
**∑PUFA ^6^**	8.558	a	7.706	b	8.182		8.081		2.119	0.0328	0.7990	0.8004
**∑n-9 ^7^**	47.316		48.147		47.489		47.974		2.520	0.0791	0.3029	0.1589
**∑n-6 ^8^**	8.175	a	7.330	b	7.815		7.690		2.077	0.0307	0.7473	0.7869
**∑n-3 ^9^**	0.280		0.281		0.269	b	0.292	a	0.053	0.9571	0.0183	0.9769
**∑n-6:∑n-3**	29.234	a	26.290	b	28.911	a	26.612	b	5.875	0.0081	0.0376	0.6738
**C18:1n-9/C18:0**	4.308		4.526		4.372		4.462		0.742	0.1169	0.5159	0.1891
**C16:1n-7/C16:0**	0.163		0.175		0.169		0.170		0.034	0.0631	0.8295	0.3960
**Δ-9-desaturase ^10^**	0.601		0.608		0.602		0.606		0.028	0.1955	0.4868	0.0910
**Δ-6-desaturase ^11^**	0.021		0.023		0.022		0.022		0.005	0.0698	0.9575	0.7869
**Δ-5-desaturase ^12^**	0.942		0.941		0.944		0.939		0.019	0.7744	0.1992	0.2422

^1^ RMSE = Root mean square error (pooled SD); ^2^ *p* = differences were statistically different when *p* < 0.05; ^3^ IMF = intramuscular fat (%); ^4^ ∑SAT = sum of total saturated fatty acids; ^5^ ∑MUFA = sum of total monounsaturated fatty acids; ^6^ ∑PUFA = sum of total polyunsaturated fatty acids; ^7^ ∑n-9 = sum of total n-9 fatty acids; ^8^ ∑n-6 = sum of total n-6 fatty acids; ^9^ ∑n-3 = sum of total n-3 fatty acids; ^10^ Δ-9 − desaturase index = (C14:1 + C16:1 + C18:1)/(C14:0 + C14:1 + C16:0 + C16:1 + C18:0 + C18:1); ^11^ Δ6-desaturase = (C18:3n-6 + C18:4n-3)/(C18:2n-6 + C18:3n-3 + C18:3n-6 + C18:4n-3); ^12^ Δ5-desaturase = (C20:4n-6)/(C20:3n-6 + C20:4n-6); ^a,b^ Letters with different superscripts were statistically significant.

## Data Availability

Data are contained within the article.

## Appendix A

**Table A1:** Analyzed composition and fatty acid proportion of the sows’ experimental diets.

*Analyzed Composition* ^1^	CONTROL	VE^2^	HXT	HXT + VE
Dry matter, %	90.8	89.8	91.1	91.2
Crude Protein, %	13.1	14.0	15.0	14.5
Fat, %	4.3	3.6	3.8	3.7
Ash, %	6.5	6.4	6.9	6.0
Fiber, %	4.3	4.4	4.3	4.4
Starch, %	49.0	46.1	41.1	43.0
Vitamin E, mg/kg	70.5	103.6	65.4	114.1
***Fatty acid composition***				
C14:0	0.60	0.66	0.56	0.60
C16:0	19.80	21.48	19.10	20.11
C16:1n-9	0.12	0.15	0.12	0.12
C16:1n-7	0.79	0.87	0.73	0.78
C18:0	4.77	4.80	4.45	4.54
C18:1n-9	28.97	25.26	30.33	26.94
C18:1n-7	1.92	1.49	1.57	1.60
C18:2n-6	38.97	40.81	38.97	40.89
C18:3n-3	3.04	3.44	3.15	3.36
C20:0	0.30	0.26	0.30	0.31
C20:1n-9	0.52	0.58	0.55	0.58
∑SAT	25.47	27.20	24.41	25.55
∑MUFA	32.33	28.35	33.29	30.02
∑PUFA	42.01	44.25	42.11	44.25

^1^ Ingredients (%): Corn: 16.03; wheat: 10; Barley: 50; soya meal 47: 6.73; wheat bran: 6.54; pork lard: 1; beetroot pulp: 5; calcium carbonate: 1.98; bicalcium phosphate: 0.54; salt: 0.5; L-lysine: 0.50; Methionine: 0.04; threonine: 0.18; choline chloride: 0.03; premix: 0.3 (per kg/diet: vitamin A: 12,000 IU; Vitamin D_3_: 1400 IU; Vitamin B_1_: 1.1 mg; Vitamin B_2_: 6 mg; Vitamin B_12_: 0.08 mg; Vitamin B_6_: 12 mg; Nicotinic acid: 21 mg; Biotin: 0.12 mg; Pantothenic acid: 12 mg; Vitamin K_3_: 1.1 mg; Choline chloride: 225 mg; Fe (ferrous carbonate): 60 mg; Cu (pentahydrate sulphate): 14.2 mg; Zn (oxide): 100 mg; Mn (monohydrate sulphate): 30 mg; I (potassium iodure): 0.8 mg; Se (sodium selenite): 0.3 mg. Calculated metabolizable energy (ME) based on ingredient composition = 3030 Kcal ME/kg.

**Table A2:** Calculated composition of the piglets’ diets during the growing period.

	10–90 Days ^1^	91–150 Days ^2^	151–240 Days ^3^
Humidity, %	10.52	11.24	10.79
Crude Protein, %	14.46	11.50	10.42
Fat, %	4.68	3.17	4.66
Fiber, %	3.71	4.53	5.71
Ash, %	4.80	4.90	5.22
Starch, %	45.55	47.19	45.57
Sugars, %	4.09	2.00	2.78
Met, %	0.31	0.25	0.20
Met+ Cys, %	0.57	0.49	0.42
Lys, %	0.98	0.81	0.65
Thr, %	0.62	0.53	0.42
Thp, %	0.70	0.15	0.11
Ca, %	0.70	0.80	1.00
P, %	0.43	0.44	0.39
ME (Kcal/kg)	3250	3100	3100

^1^ Ingredients (%): Corn: 25; wheat: 20; barley: 30; fish protein: 5; soya 47: 8.71; beetroot pulp: 3; milk serum: 2.5; monocalcium phosphate: 0.42; calcium carbonate: 0.96; lard: 2.72; trn: 0.1; lys: 0.5; met: 0.06; salt: 0.3; enzymes: 0.1; protacid (60% formic acid + 40% lignosulfonic acid): 0.3; choline chloride: 0.02; premix: 0.3. ^2^ Ingredients (%): Corn: 23.6; wheat: 10; barley: 50; wheat brand: 3.6; soya 47: 5.17; beetroot pulp: 3; monocalcium phosphate: 0.55; calcium carbonate: 1.49; lard: 1; trp: 0.08; trn: 0.15; lys: 0.62; met: 0.06; salt: 0.4; premix: 0.3. ^3^ Ingredients (%): Corn: 28.4; wheat: 10; barley: 41.4; beet molasses: 2; sunflower 28: 2.72; beetroot pulp: 5; high oleic sunflower seeds: 6.8; monocalcium phosphate: 0.42; calcium carbonate: 1.96; Trn: 0.09; lys: 0.53; met: 0.01; salt: 0.4; premix: 0.3.
