# Correction: Gómez et al. Long-Term Effect of Maternal Antioxidant Supplementation on the Lipid Profile of the Progeny According to the Sow’s Parity Number. *Antioxidants* 2024, *13*, 379

**DOI:** 10.3390/antiox13080987

**Published:** 2024-08-14

**Authors:** Gerardo Gómez, Hernan D. Laviano, Juan García-Casco, Maria Muñoz, Fernando Gómez, Fernando Sánchez-Esquiliche, Antonio González-Bulnes, Clemente López-Bote, Cristina Óvilo, Ana I. Rey

**Affiliations:** 1 Instituto Regional de Investigación y Desarrollo Agroalimentario y Forestal de Castilla-La Mancha (IRIAF), 13700 Toledo, Spain; g.gomez.mat@gmail.com; 2 Departamento Producción Animal, Facultad de Veterinaria, Universidad Complutense de Madrid, Avda. Puerta de Hierro s/n., 28040 Madrid, Spain; 3 Departamento de Mejora Genética Animal, Instituto Nacional de Investigación y Tecnología Agraria y Alimentaria (INIA), Consejo Superior de Investigaciones Científicas (CSIC), Ctra Coruña km 7.5, 28040 Madrid, Spain; 4 Sánchez Romero Carvajal, Carretera de San Juan del Puerto, s/n, 21290 Jabugo, Spain; 5 Departamento de Producción y Sanidad Animal, Facultad de Veterinaria, Universidad Cardenal Herrera-CEU, CEU Universities, C/Tirant lo Blanc, 7, Alfara del Patriarca, 46115 Valencia, Spain

## 1. Error in Table 2

In the original publication [1], there was a mistake in Table 2 as published. The percentage of C16:1n-7 fatty acid was missing in Table 2, but this was discussed in the text. The corrected Table 2 appears below.

## 2. Error in Table A2

In the original publication [1], there was a mistake in Table A2 as published. The dry matter % of piglets’ diets in Table A2 should be Humidity %.

The authors state that the scientific conclusions are unaffected. This correction was approved by the Academic Editor. The original publication has also been updated.

## Figures and Tables

**Table 2:** Effect of maternal supplementation with VITE (30 vs. 100 mg α-tocopherol acetate/kg) or HXT (0 vs. 1.5 mg/kg) on the total fatty acid profile of intramuscular fat of the longissimus dorsi muscle (%) from Iberian pigs at slaughter (15 months of age) after free-range fattening.

	VITE-30		VITE-100		HXT-0		HXT-1.5		RMSE ^1^	*p* VITE ^2^	*p* HXT	*p* VITEXHXT
**IMF ^3^**	12.842		12.151		12.727		12.266		6.216	0.5518	0.6912	0.9136
**C14:0**	1.465		1.476		1.447		1.493		0.257	0.8241	0.3406	0.1426
**C15:0**	0.034		0.033		0.033		0.034		0.011	0.8050	0.5973	0.9736
**C16:0**	22.628		22.777		22.862		22.544		2.058	0.6969	0.4078	0.2493
**C16:1n-9**	0.257	a	0.237	b	0.245		0.249		0.046	0.0246	0.6141	0.0846
**C16:1n-7**	3.691	b	3.967	a	3.853		3.805		0.680	0.0311	0.7045	0.7540
**C17:0**	0.163		0.157		0.155		0.164		0.038	0.3931	0.2039	0.6529
**C17:1**	0.244		0.245		0.250		0.239		0.071	0.9098	0.4183	0.9598
**C18:0**	10.920		10.589		10.718		10.790		1.285	0.1685	0.7653	0.1592
**C18:1n-9**	46.239		47.077		46.425		46.892		2.468	0.0706	0.3116	0.1830
**C18:1n-7**	4.827		4.754		4.859		4.722		0.855	0.6445	0.3914	0.7541
**C18:2n-6**	6.167	a	5.498	b	5.832		5.834		1.716	0.0384	0.9947	0.8210
**C18:3n-6**	0.045		0.041		0.044		0.042		0.016	0.1398	0.5532	0.9296
**C18:3n-3**	0.195		0.194		0.185	b	0.205	a	0.050	0.9097	0.0372	0.7318
**C18:4n-3**	0.085		0.087		0.084		0.088		0.016	0.5937	0.1830	0.2434
**C20:0**	0.156		0.151		0.152		0.154		0.031	0.3786	0.7754	0.3473
**C20:1n-9**	0.820		0.832		0.819		0.833		0.101	0.5210	0.4487	0.0663
**C20:2n-6**	0.212		0.200		0.199		0.213		0.051	0.2072	0.1452	0.6099
**C20:3n-6**	0.102		0.095		0.098		0.098		0.028	0.1659	0.9285	0.3311
**C20:4n-6**	1.750		1.591		1.740		1.601		0.507	0.0936	0.1433	0.7696
**∑SAT ^4^**	35.365		35.182		35.368		35.179		2.709	0.7177	0.7082	0.0924
**∑MUFA ^5^**	56.078	b	57.112	a	56.450		56.740		2.774	0.0474	0.5757	0.1462
**∑PUFA ^6^**	8.558	a	7.706	b	8.182		8.081		2.119	0.0328	0.7990	0.8004
**∑n-9 ^7^**	47.316		48.147		47.489		47.974		2.520	0.0791	0.3029	0.1589
**∑n-6 ^8^**	8.175	a	7.330	b	7.815		7.690		2.077	0.0307	0.7473	0.7869
**∑n-3 ^9^**	0.280		0.281		0.269	b	0.292	a	0.053	0.9571	0.0183	0.9769
**∑n-6:∑n-3**	29.234	a	26.290	b	28.911	a	26.612	b	5.875	0.0081	0.0376	0.6738
**C18:1n-9/C18:0**	4.308		4.526		4.372		4.462		0.742	0.1169	0.5159	0.1891
**C16:1n-7/C16:0**	0.163		0.175		0.169		0.170		0.034	0.0631	0.8295	0.3960
**Δ-9-desaturase ^10^**	0.601		0.608		0.602		0.606		0.028	0.1955	0.4868	0.0910
**Δ-6-desaturase ^11^**	0.021		0.023		0.022		0.022		0.005	0.0698	0.9575	0.7869
**Δ-5-desaturase ^12^**	0.942		0.941		0.944		0.939		0.019	0.7744	0.1992	0.2422

^1^ RMSE = Root mean square error (pooled SD); ^2^ *p* = differences were statistically different when *p* < 0.05; ^3^ IMF = intramuscular fat (%); ^4^ ∑SAT = sum of total saturated fatty acids; ^5^ ∑MUFA = sum of total monounsaturated fatty acids; ^6^ ∑PUFA = sum of total polyunsaturated fatty acids; ^7^ ∑n-9 = sum of total n-9 fatty acids; ^8^ ∑n-6 = sum of total n-6 fatty acids; ^9^ ∑n-3 = sum of total n-3 fatty acids; ^10^ Δ-9 − desaturase index = (C14:1 + C16:1 + C18:1)/(C14:0 + C14:1 + C16:0 + C16:1 + C18:0 + C18:1); ^11^ Δ6-desaturase = (C18:3n-6 + C18:4n-3)/(C18:2n-6 + C18:3n-3 + C18:3n-6 + C18:4n-3); ^12^ Δ5-desaturase = (C20:4n-6)/(C20:3n-6 + C20:4n-6); ^a,b^ Letters with different superscripts were statistically significant.

**Table A2:** Calculated composition of the piglets’ diets during the growing period.

	10–90 Days ^1^	91–150 Days ^2^	151–240 Days ^3^
Humidity, %	10.52	11.24	10.79
Crude Protein, %	14.46	11.50	10.42
Fat, %	4.68	3.17	4.66
Fiber, %	3.71	4.53	5.71
Ash, %	4.80	4.90	5.22
Starch, %	45.55	47.19	45.57
Sugars, %	4.09	2.00	2.78
Met, %	0.31	0.25	0.20
Met+ Cys, %	0.57	0.49	0.42
Lys, %	0.98	0.81	0.65
Thr, %	0.62	0.53	0.42
Thp, %	0.70	0.15	0.11
Ca, %	0.70	0.80	1.00
P, %	0.43	0.44	0.39
ME (Kcal/kg)	3250	3100	3100

^1^ Ingredients (%): Corn: 25; wheat: 20; barley: 30; fish protein: 5; soya 47: 8.71; beetroot pulp: 3; milk serum: 2.5; monocalcium phosphate: 0.42; calcium carbonate: 0.96; lard: 2.72; trn: 0.1; lys: 0.5; met: 0.06; salt: 0.3; enzymes: 0.1; protacid (60% formic acid + 40% lignosulfonic acid): 0.3; choline chloride: 0.02; premix: 0.3. ^2^ Ingredients (%): Corn: 23.6; wheat: 10; barley: 50; wheat brand: 3.6; soya 47: 5.17; beetroot pulp: 3; monocalcium phosphate: 0.55; calcium carbonate: 1.49; lard: 1; trp: 0.08; trn: 0.15; lys: 0.62; met: 0.06; salt: 0.4; premix: 0.3. ^3^ Ingredients (%): Corn: 28.4; wheat: 10; barley: 41.4; beet molasses: 2; sunflower 28: 2.72; beetroot pulp: 5; high oleic sunflower seeds: 6.8; monocalcium phosphate: 0.42; calcium carbonate: 1.96; Trn: 0.09; lys: 0.53; met: 0.01; salt: 0.4; premix: 0.3.
